# Supplementary figures and images for: 7-Dehydrocholesterol-derived oxysterols cause neurogenic defects in Smith-Lemli-Opitz syndrome
Source: eLife. 2022 Sep 16;11:e67141. doi: 10.7554/eLife.67141 (PMC9519149; doi:10.7554/eLife.67141)

**E11.5 E13.5 E15.5 E17.5 P0**

**Dhcr7**

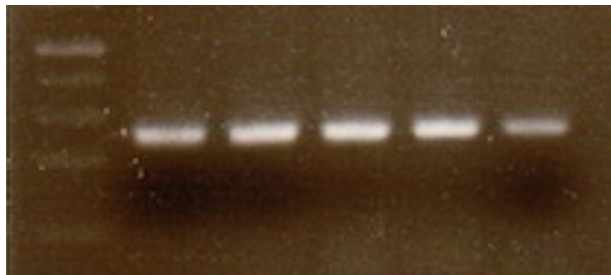

**Actin**

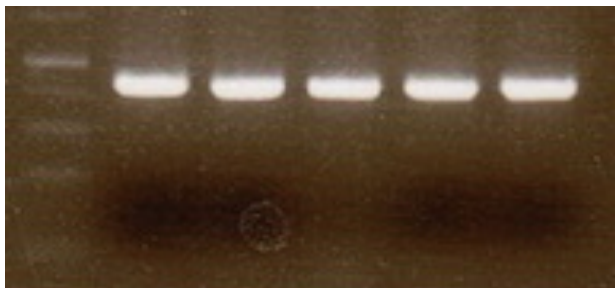

Supplement: Figure 1—source data 1. — RT-PCR for Dhcr7 mRNA in the E11.5 to P0 cortex. β-actin mRNA was used as loading control. [file elife-67141-fig1-data1.pdf]

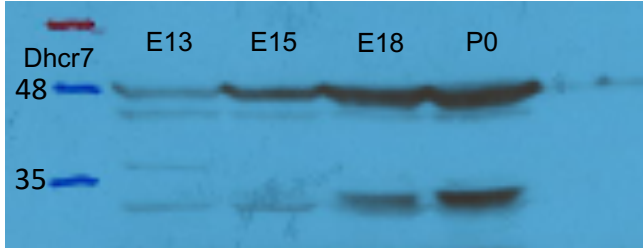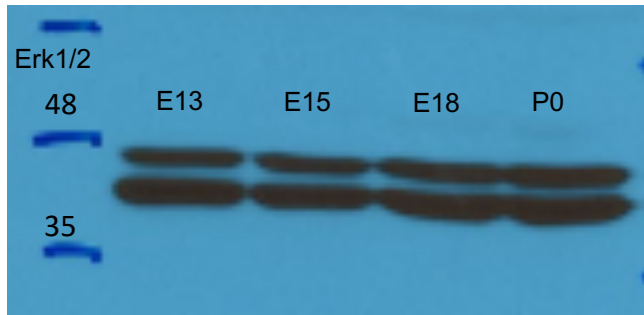

Supplement: Figure 1—source data 2. — Western blot of Dhcr7 in total cortical lysates from E13.5 to P0. The blot was re-probed for Erk1/2 as a loading control. [file elife-67141-fig1-data2.pdf]

WT

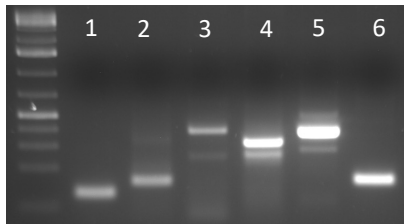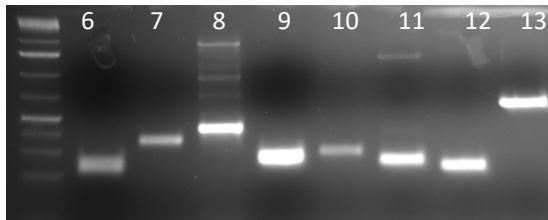

3044

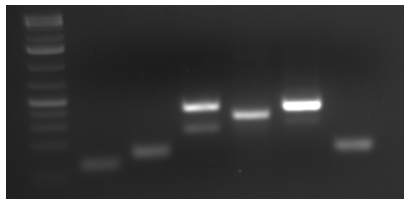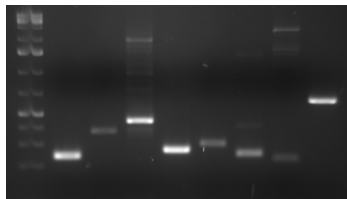

5788

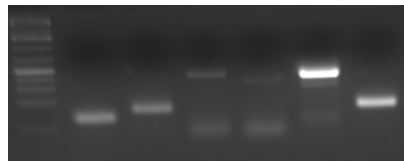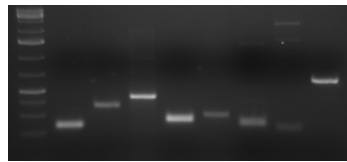

Supplement: Figure 1—figure supplement 1—source data 1. — RT-PCR analysis of hES cell marker genes in human iPSCs derived from SLOS patients (3044 and 5788) and healthy individual (emhf2). [file elife-67141-fig1-figsupp1-data1.pdf]

mDhcr7  
KD

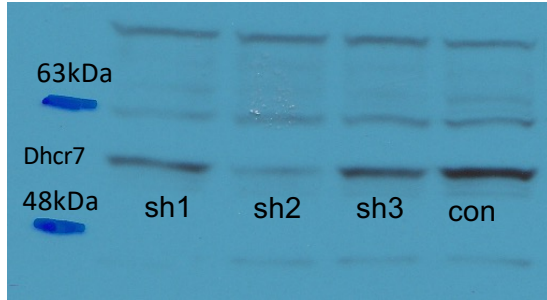

Erk1/2

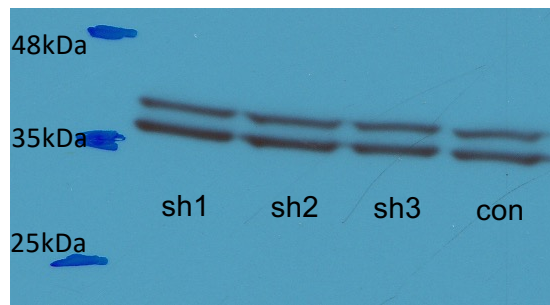

Supplement: Figure 3—source data 1. — Western blot for Dhcr7 in 293T cells transfected with control or individual murine Dhcr7 shRNAs. The blot was re-probed for Erk1/2 as a loading control. [file elife-67141-fig3-data1.pdf]

hDHCR7  
KD

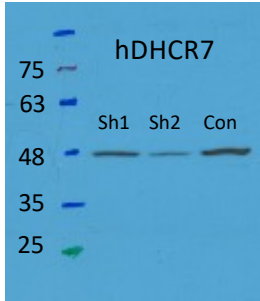

Erk1/2

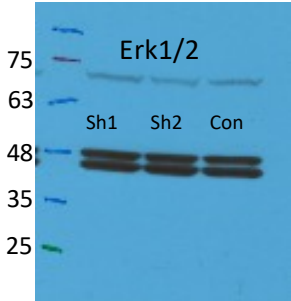

Supplement: Figure 3—source data 2. — Western blots of DHCR7 in 293T cells transfected with human control (Con) or human-specific DHCR7 shRNA (sh3) plus human DHCR7-expressing plasmid, analyzed after 24 hr. The blot was re-probed for Erk1/2. [file elife-67141-fig3-data2.pdf]

*Dhcr7*  
E15.5 CTX

+/+ -/-

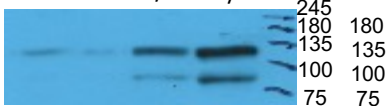

pTrkB

+/+ -/-

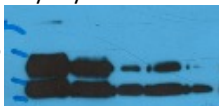

TrkB

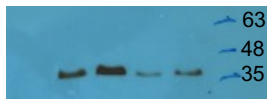

pMEK

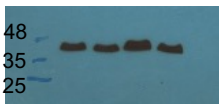

MEK

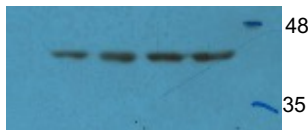

pC/EBP

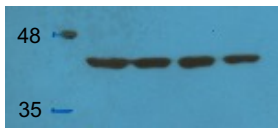

C/EBP

+/+ -/-

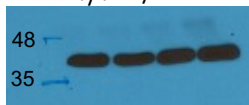

GAPDH

Supplement: Figure 5—source data 1. — E15.5 cortices were isolated from Dhcr7+/+ and Dhcr7-/- embryos and analyzed by western blot for phospho-TrkB, phospho-MEK, or phospho-C/EBPβ. Blots were re-probed with antibodies for total GR, TrkB, MEK, C/EBPβ, and GAPDH as loading controls. [file elife-67141-fig5-data1.pdf]

*Dhcr7*  
E15.5 CTX

---

+/+

+/+

-/-

pGR

135

100

75

GR

135

100

GAPDH

48

35

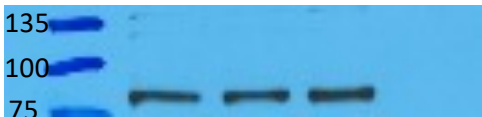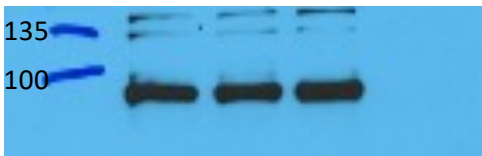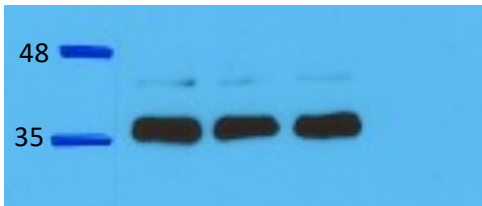

Supplement: Figure 8—source data 1. — Western blot showing increased phospho-GR in E15.5 Dhcr7-/- mouse brain relative to Dhcr7+/+. [file elife-67141-fig8-data1.pdf]

DHCEO (hrs)

pGR

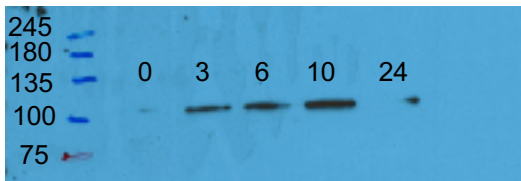

GR

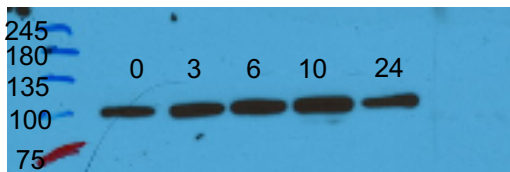

pTrkB

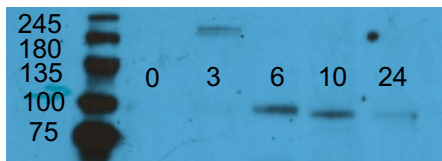

TrkB

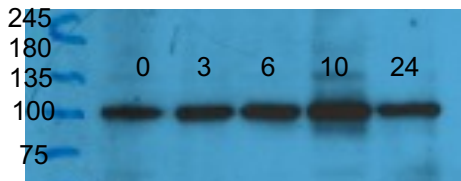

Erk1/2

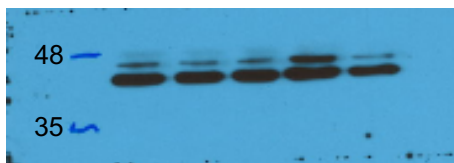

Supplement: Figure 8—source data 2. — Human neural progenitors were treated with 3.5 μM DHCEO over the indicated time periods. Lysates were probed with phosphor-GR and phosphor-TrkB and re-probed with antibodies for total GR, total TrkB or total ERK as loading controls. [file elife-67141-fig8-data2.pdf]

PD98059

Trametinib

+

-

-

+

pMEK

48

35

25

MEK

48

35

25

GAPDH

48

35

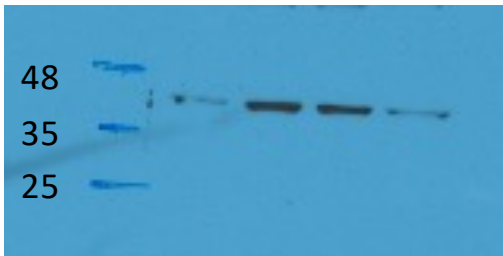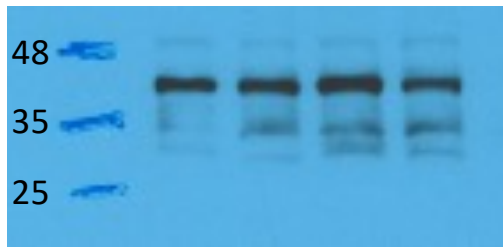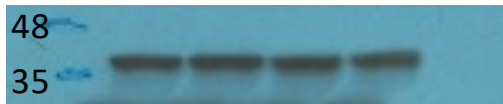

Supplement: Figure 8—source data 3. — hNPCs were treated or not treated with MEK/ERK inhibitors, trametinib or PD98059. Western blot of phosphor-MEK. The blots were then re-probed with antibodies for total MEK as loading controls. [file elife-67141-fig8-data3.pdf]

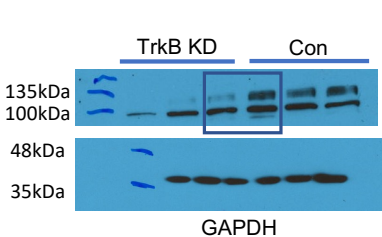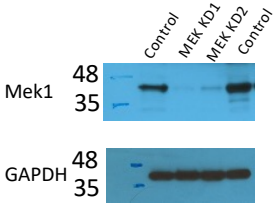

Supplement: Figure 8—source data 4. — Western blots for TrkB or MEK1/2 in lysate of 293T cells transfected with control or TrkB shRNA or MEK shRNA vector. The blots were re-probed for glyceraldehyde 3-phosphate dehydrogenase (GAPDH). [file elife-67141-fig8-data4.pdf]
