# Supplementary material for: 7-Dehydrocholesterol-derived oxysterols cause neurogenic defects in Smith-Lemli-Opitz syndrome
Source: eLife. 2022 Sep 16;11:e67141. doi: 10.7554/eLife.67141 (PMC9519149; doi:10.7554/eLife.67141)
Supplement: Figure 1—figure supplement 1—source data 2. — RT-PCR analyses and Western blot analyses of differentiation markers for the three germ layers. [file elife-67141-fig1-figsupp1-data2.pdf]

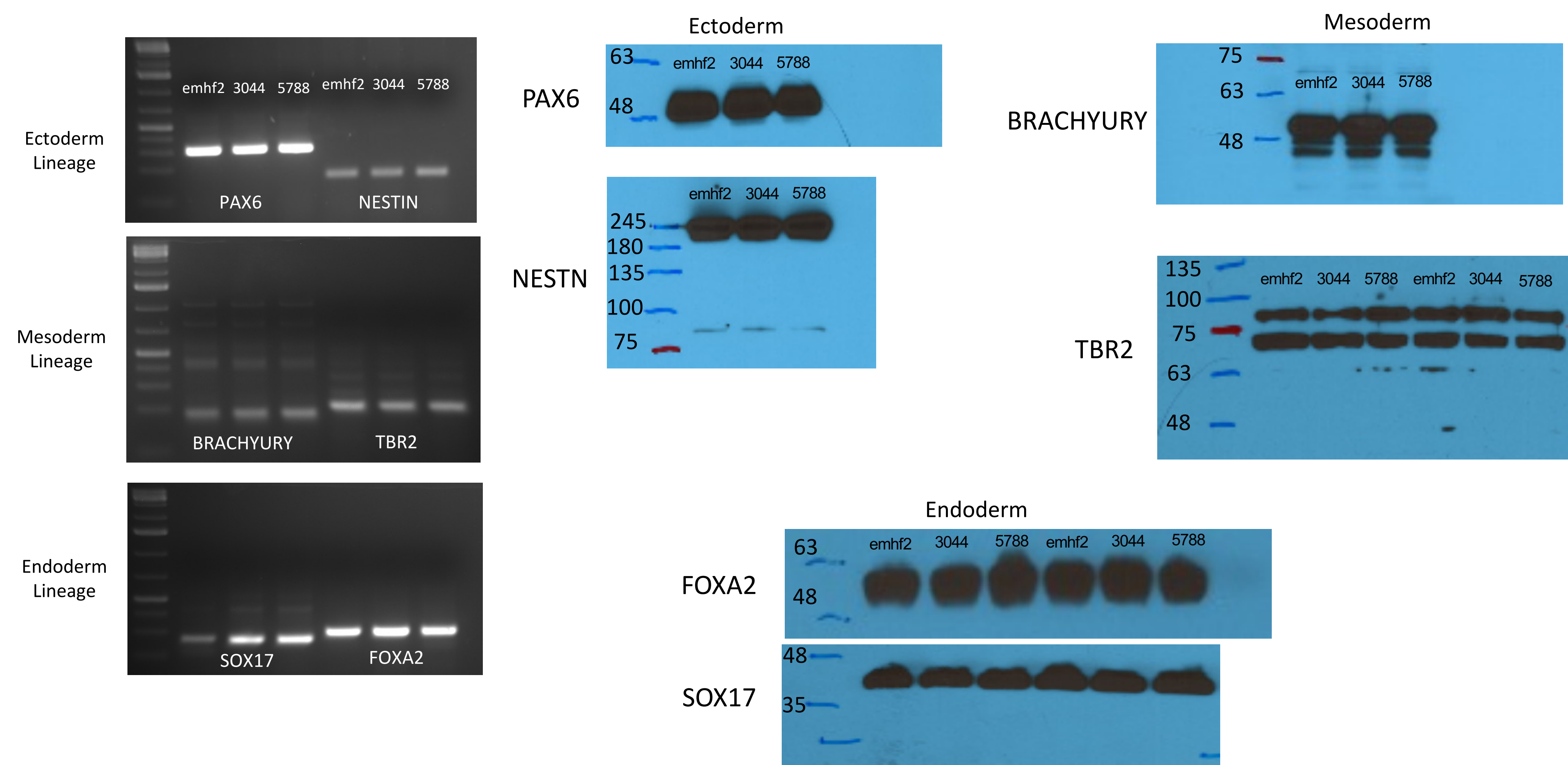

Ectoderm Lineage
emhf2 3044 5788 emhf2 3044 5788
PAX6
NESTIN
Mesoderm Lineage
BRACHYURY
TBR2
Endoderm Lineage
SOX17
FOXA2
Ectoderm
PAX6
63
48
emhf2 3044 5788
NESTN
emhf2 3044 5788
245
180
135
100
75
Mesoderm
BRACHYURY
75
63
48
emhf2 3044 5788
TBR2
135
100
75
63
48
emhf2 3044 5788 emhf2 3044 5788
Endoderm
FOXA2
63
48
emhf2 3044 5788 emhf2 3044 5788
SOX17
48
35
