# Supplementary material for: 7-Dehydrocholesterol-derived oxysterols cause neurogenic defects in Smith-Lemli-Opitz syndrome
Source: eLife. 2022 Sep 16;11:e67141. doi: 10.7554/eLife.67141 (PMC9519149; doi:10.7554/eLife.67141)
Supplement: Supplementary file 1. — Relate to Figure 2 and Figure 2-Figure Supplement 2. [file elife-67141-supp1.docx]

**Supplementary File 1. Retention times and MS/MS transitions for oxysterol internal standards. Relate to Figure 2 and Figure 2-Figure Supplement 2.**

| **Internal Standard** | **Retention**  **Time (min)** | **Q1** | **Q3** |
| --- | --- | --- | --- |
| d_7_-DHCEO | 1.80 | 424.3 | 406.3 |
| d_7_-7-ketocholesterol | 2.91 | 408.3 | 390.3 |
| d_6_-24,25-epoxycholesterol | 1.94 | 389.3 | 371.3 |
| d_7_-24-hydroxycholesterol | 1.63 | 392.3 | 374.3 |
| d_7_-4β-hydroxycholesterol | 5.16 | 392.3 | 374.3 |
