# Supplementary material for: 7-Dehydrocholesterol-derived oxysterols cause neurogenic defects in Smith-Lemli-Opitz syndrome
Source: eLife. 2022 Sep 16;11:e67141. doi: 10.7554/eLife.67141 (PMC9519149; doi:10.7554/eLife.67141)
Supplement: Supplementary file 2. — Relate to Figure 2 and Figure 2—figure supplement 2. [file elife-67141-supp2.docx]

**Supplementary File 2. Retention times and MS/MS transitions for all oxysterol standards. Relate to Figure 2 and Figure 2-Figure Supplement 2.**

| **Analyte** | **Retention**  **Time (min)** | **Q1** | **Q3** | **Std Conc. Std+IS Mix** | **IS Used** | **IS Conc. Std+IS Mix** |
| --- | --- | --- | --- | --- | --- | --- |
| 24-hydroxycholesterol | 1.63 | 385.3 | 367.3 | 0.2 ug/mL | d_7_-24-hydroxycholesterol | 0.2 ug/mL |
| 25-hydroxycholesterol | 1.63 | 385.3 | 367.3 | 0.2 ug/mL | d_7_-24-hydroxycholesterol | 0.2 ug/mL |
| 24-ketocholesterol | 1.79 | 383.3 | 365.3 | 0.2 ug/mL | d_6_-24,25-epoxycholesterol | 0.2 ug/mL |
| 24-epoxycholesterol | 1.94 | 383.3 | 365.3 | 0.2 ug/mL | d_6_-24,25-epoxycholesterol | 0.2 ug/mL |
| 7β-hydroxycholesterol | 2.65 | 385.3 | 367.3 | 0.2 ug/mL | d_7_-7-ketocholesterol | 0.2 ug/mL |
| 7α-hydroxycholesterol | 2.65 | 385.3 | 367.3 | 0.2 ug/mL | d_7_-7-ketocholesterol | 0.2 ug/mL |
| 7-ketocholesterol | 2.96 | 401.3 | 383.3 | 0.2 ug/mL | d_7_-7-ketocholesterol | 0.2 ug/mL |
| 4β-hydroxycholesterol | 5.16 | 385.3 | 367.3 | 0.2 ug/mL | d_7_-4β-hydroxycholesterol | 0.2 ug/mL |
| 24-hydroxy-7-DHC | 1.43 | 383.3 | 365.3 | 0.2 ug/mL | d_7_-DHCEO | 0.2 ug/mL |
| DHCEO | 1.83 | 417.3 | 399.3 | 0.2 ug/mL | d_7_-DHCEO | 0.2 ug/mL |
| 7-keto-DHC | 2.14 | 399.3 | 381.3 | 0.2 ug/mL | d_7_-DHCEO | 0.2 ug/mL |
| 4α-hydroxy-7-DHC | 3.77 | 365.3 | 365.3 | 0.2 ug/mL | d_7_-DHCEO | 0.2 ug/mL |
| 4β-hydroxy-7-DHC | 4.37 | 365.3 | 365.3 | 0.2 ug/mL | d_7_-DHCEO | 0.2 ug/mL |
