# Supplementary material for: 7-Dehydrocholesterol-derived oxysterols cause neurogenic defects in Smith-Lemli-Opitz syndrome
Source: eLife. 2022 Sep 16;11:e67141. doi: 10.7554/eLife.67141 (PMC9519149; doi:10.7554/eLife.67141)
Supplement: Supplementary file 3. — Relate to Figure 2 and Figure 2—figure supplement 2. [file elife-67141-supp3.docx]

**Supplementary File 3. Retention times and MS/MS transitions for sterol internal standards. Relate to Figure 2 and Figure 2-Figure Supplement 2.**

| **Internal Standard** | **Retention**  **Time (min)** | **Q1** | **Q3** |
| --- | --- | --- | --- |
| ^13^C_3_-desmosterol | 6.40 | 370.3 | 370.3 |
| d_7_-7-dehydrocholesterol | 6.90 | 374.3 | 374.3 |
| d_7_-cholesterol | 8.60 | 376.3 | 376.3 |
| ^13^C_3_-lanosterol | 9.83 | 412.3 | 412.3 |
