# Supplementary material for: 7-Dehydrocholesterol-derived oxysterols cause neurogenic defects in Smith-Lemli-Opitz syndrome
Source: eLife. 2022 Sep 16;11:e67141. doi: 10.7554/eLife.67141 (PMC9519149; doi:10.7554/eLife.67141)
Supplement: Supplementary file 4. — Relate to Figure 2 and Figure 2—figure supplement 2. [file elife-67141-supp4.docx]

**Supplementary File 4. Retention times and MS/MS transitions for sterol standards. Relate to Figure 2 and Figure 2-Figure Supplement 2.**

| **Analyte** | **Retention**  **Time (min)** | **Q1** | **Q3** | **Std Conc. Std+IS Mix** | **IS Used** | **IS Conc. Std+IS Mix** |
| --- | --- | --- | --- | --- | --- | --- |
| 7-Dehydrodesmosterol | 5.18 | 365.3 | 365.3 | 0.4 ug/mL | ^13^C_3_-desmosterol | 0.4 ug/mL |
| Zymosterol | 6.01 | 367.3 | 367.3 | 0.4 ug/mL | ^13^C_3_-desmosterol | 0.4 ug/mL |
| Desmosterol | 6.38 | 367.3 | 367.3 | 0.4 ug/mL | ^13^C_3_-desmosterol | 0.4 ug/mL |
| 8-Dehydrocholesterol | 6.79 | 367.3 | 367.3 | 0.4 ug/mL | d_7_-7-dehydrocholesterol | 2.0 ug/mL |
| 7-Dehydrocholesterol | 7.0 | 367.3 | 367.3 | 0.4 ug/mL | d_7_-7-dehydrocholesterol | 2.0 ug/mL |
| Lathosterol | 8.28 | 369.3 | 369.3 | 0.4 ug/mL | ^13^C_3_-lanosterol | 0.4 ug/mL |
| Cholesterol | 8.69 | 369.3 | 369.3 | 0.4 ug/mL | d_7_-cholesterol | 2.0 ug/mL |
| Lanosterol | 9.86 | 409.3 | 409.3 | 0.4 ug/mL | ^13^C_3_-lanosterol | 0.4 ug/mL |
