# Supplementary material for: 7-Dehydrocholesterol-derived oxysterols cause neurogenic defects in Smith-Lemli-Opitz syndrome
Source: eLife. 2022 Sep 16;11:e67141. doi: 10.7554/eLife.67141 (PMC9519149; doi:10.7554/eLife.67141)
Supplement: Supplementary file 5. — Relate to Figure 7. DEGs were further analyzed with (IPA) to identify the most enriched biological functions related to the nervous system in SLOS mutant NPCs. The table below shows the top ten enriched terms corresponding to Diseases/Bio-functions along with the p-value and overlapping number of genes in the dataset. [file elife-67141-supp5.docx]

**Supplementary File 5. Ingenuity Pathway Analysis (IPA®) reveals “development of the central nervous system” as one of the top 10 enriched Diseases and Biological Functions related to the nervous system.** DEGs were further analyzed with (IPA®) to identify the most enriched biological functions related to the nervous system in SLOS mutant NPCs. The table below shows the top ten enriched terms corresponding to Diseases/Bio-functions along with the *p*-value and overlapping number of genes in the dataset.

| **Disease or Function** | **p-Value** | **# genes in dataset** |
| --- | --- | --- |
| Development of neurons | 1.59E-47 | 275 |
| Morphology of nervous system | 4.71E-47 | 311 |
| Abnormal morphology of nervous system | 7.45E-42 | 265 |
| Development of sensory organ | 9.01E-33 | 186 |
| Development of central nervous system | 3.28E-29 | 199 |
| Neurotransmission | 6.97E-29 | 143 |
| Abnormal morphology of central nervous system | 1.42E-28 | 162 |
| Morphogenesis of neurons | 1.53E-28 | 192 |
| Neuritogenesis | 5.85E-28 | 189 |
| Morphology of central nervous system | 7.02E-28 | 184 |
